# Infectious Wildlife Diseases in Austria—A Literature Review From 1980 Until 2017

**DOI:** 10.3389/fvets.2020.00003

**Published:** 2020-02-21

**Authors:** Nina Eva Trimmel, Chris Walzer

**Affiliations:** ^1^Department of Integrative Biology and Evolution, Research Institute of Wildlife Ecology, University of Veterinary Medicine Vienna, Vienna, Austria; ^2^Wildlife Conservation Society, Bronx, NY, United States

**Keywords:** wildlife, infectious, diseases, zoonosis, Austria, game, one health, EID

## Abstract

This literature review examines infectious wildlife disease research in Austria. We analyzed 226 research papers, published between 1980 and 2017. We determined that wildlife disease papers increased significantly from 0.8 ± 0.8 publications per year in the first decade (1980–1989) when compared to 2008–2017 with an average of 12.9 ± 4.1 publications per year. We illustrate information about the most investigated diseases and highlight the lack of research into certain wildlife pathogens. A special emphasis was given to diseases with zoonotic potential. The review showed that research focused on a few select species like the red fox (*Vulpes vulpes*), red deer (*Cervus elaphus*), and wild boar (*Sus scrofa*), all game species. Moreover, diseases affecting livestock and human health were seen more often. The review also found that only a low number of publications actually stated disease prevalence and confidence interval data. The reported diseases identified were classified according to their notifiable status and the distribution at the wildlife–human and wildlife–livestock interface. Furthermore, we try to argue why research into some diseases is prioritized, and why other diseases are underrepresented in current Austrian research. While spatiotemporal indicators could not be assessed due to the variability in methodologies and objectives of various studies, the information provided by this review offers the first comprehensive evaluation of the status of infectious wildlife disease research in Austria. Therefore, this study could assist investigators to identify further areas of priorities for research and conservation efforts and for wildlife management professionals to inform policy and funding strategies. With this review, we want to encourage research in the field of wildlife diseases in Austria to enhance current knowledge in the prevention of further loss in biodiversity and to find new measures to promote “One Health” on a global scale.

## Introduction

We determined the status of infectious wildlife disease research in Austria based on a 37-year literature review between 1980 and 2017. The literature review was modeled on a similar study from the Republic of Korea in 2017 (1). One reason for conducting this review was to determine if an increase in so-called emerging infectious diseases (EIDs) had been noted in Austrian wildlife. It has previously been determined that that ~70% of EIDs have their origin in wildlife, and an estimated 60–80% of all EIDs also have zoonotic potential (2, 3). Increases in EIDs are related to the human impacts on landscapes (3, 4). In our constantly changing world, ecosystems continuously transform and offer pathogens novel opportunities to evolve, resulting in a greater diversity of adaptable and rapidly spreading infectious agents (5). Pathogens that can infect several species of hosts, so-called multihost pathogens, can also represent cause for concern with regard to wildlife conservation (6). Wildlife potentially contributes to the spread and maintenance of infectious diseases, and knowing the status supports addressing and mitigating impacts.

Austria has a population of ~8.8 million people (2017) with an average population density of 105 people/km^2^. The gross domestic product per capita is 37,100 euros (2016), which makes Austria one of the wealthiest countries in the European union (EU). A member of the European Union (EU) since 1995, the federal state of Austria is located in Central Europe and divided into nine provinces. Austria is a landlocked country with a land area of 83,879 km^2^ and a diversity of landscapes. About two-thirds of the land mass are mountainous, encompassing the Alps, the Carpathians, as well as the gneiss and granite highlands of the Bohemian Massif. The remaining third consists of the Vienna Basin and the Pannonian border regions of the Hungarian lowlands (7–9). The Austrian Fauna consists of roughly 45,870 different species, of which 626 are vertebrates. One hundred and ten vertebrates belong to the mammalian class, 418 to the avian class, 16 to the class of reptiles, 21 are amphibian species, and 60 species are fish (10).

Both active and passive surveillance programs for wildlife diseases exist in Austria (11, 12). Control programs were successful in eradicating rabies using widespread oral immunization of red fox (*Vulpes vulpes*). The last known rabies case occurred in 2003, and in 2008, Austria was declared rabies free. As of 2013, indicator animals (e.g., fox, badger, racoon dog, and raccoon) opportunistically were found dead, and all animals suspected of being rabid are examined at the National Reference Laboratory. Additional epizootic monitoring programs for Aujesky disease, bovine spongiform encephalopathy (BSE), scrapie, bluetongue virus, *Brucella melitensis*, bovine brucellosis, enzootic bovine leucosis, infectious bovine rhinotracheitis and infectious pustular vulvovaginitis, classical swine fever, and avian influenza are implemented. Only one zoonotic disease in wildlife is routinely monitored, namely, trichinosis in wild boars (*Sus scrofa*). In addition, in livestock animals, campylobacteriosis, echinococcosis, salmonellosis, trichinosis, and verotoxin-producing *Escherichia coli* are routinely monitored (13).

The wildlife-human interface in Austria consists of a number of critical points, such as game meat consumption, the hunting industry (e.g., direct contact between hunters, hunting dogs, and game animals) and tourism (e.g., hikers and recreational athletes). Occupations that involve a constant exposure to animals are related to a higher risk of infection, and this risk may be intensified through contact with livestock sharing pastures with wildlife. Risk groups include farmers, veterinarians, hunters, forestry workers, veterinary technicians, animal keepers, taxidermists, zookeepers, and people who prepare or consume game meat (14–18).

Wildlife diseases are often not included in epidemiological reflections, as the main focus remains on diseases in livestock and transmission to humans. It is important to note that the wildlife-livestock interface is bidirectional and dynamic with frequent pathogen exchange through shared resources, such as habitats, water, and feed (19, 20). These factors increase the risk of transmitting infectious diseases from wildlife to domestic animals and subsequently to humans (19). Our review serves not only as an overview but also as a reminder that research on wildlife diseases is important and must be encouraged. This review is the first and only comprehensive review of infectious wildlife diseases in Austria.

## Materials and Methods

**Figure 1:**
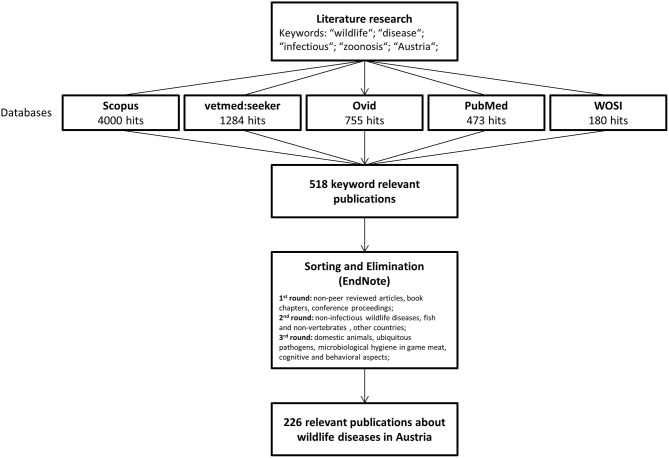
Overview of the work steps for drafting this review.

A detailed literature search was conducted between October 2018 and December 2018 using the following literature data bases: “vetmedseeker” (vetmedseeker, University of Veterinary Medicine Vienna, Austria), “Scopus” (Scopus, Elsevier, Netherlands), “Ovid” (“Ovid Technologies,” Wolters Kluwer, Netherlands), “Web of Science” (Web of Science Core Collection, Clarivate Analytics, United States), and “PubMed” (NCBI Pubmed, National Center for Biotechnology Information, United States). Search terms used included “wildlife,” “diseases,” “infectious,” and “zoonosis” adjusted by setting the time frame to 1980–2017 and the affiliation to Austria. This resulted in 2,696 hits on “Scopus,” 1,284 hits on the “vetmedseeker,” 755 on “Ovid,” 473 on “PubMed,” and 180 on “Web of Science.” Only articles published in peer-reviewed journals were retained. Papers that addressed wildlife-human or wildlife-domestic disease transmission were also included. Additional articles found manually from the respective citations were included as well. A schematic step-by-step of the literature search can be found in Figure 1.

Following abstract review, 518 publications from “Scopus” appeared relevant. Papers were then compiled using a reference managing software (End Note Version X7.8, Thomson Reuters, United States). Duplicates, non-peer reviewed articles, book chapters, and conference proceedings were excluded in a first round. In a second round, we focused on infectious pathogens and diseases occurring in wild species. We excluded papers describing non-infectious, autoimmune, or idiopathic diseases. In addition, we restricted the search to vertebrates. Multicountry studies were only included in our review if “Austria” was mentioned in the materials and methods. In a third round, we excluded publications that addressed microbiological hygiene aspects in game meat. Subsequent to this process, it was clear that “Scopus” was the most efficient database for wildlife diseases, revealing 219 out of a total of 226 relevant publications. “PubMed,” in contrast, only showed 76 relevant articles. Finally, from all databases surveyed, 226 papers were retained and entered into a Microsoft Excel file for descriptive statistical analysis.

Publications were classified according to definitions by “Springer Nature” concerning article type (21) into original research articles, reviews, case reports, and short reports or letters. Original research articles were further subdivided into controlled studies and retrospective studies. Subsequently papers were categorized according to year of publication, type of pathogen, affected animal species, and group interface. The classification of group interfaces describes the primary group of interest (e.g., wildlife, humans, or livestock animals). Furthermore, we recorded whether the disease was notifiable according to the OIE List of notifiable diseases or the current Austrian legislation. If the paper covered various infectious agents, it was assigned to the categories “Notifiable in Austria” or “OIE-listed disease,” if at least one notifiable disease was included. In addition, it was noted if the prevalence and confidence intervals of the pathogen(s) were stated in the article.

Infectious agents were divided into parasitic, viral, bacterial, and fungal pathogens, and articles covering more than one pathogen or addressing more than one species were classified as “multiple infectious agents” or “multiple species affected.” Furthermore, agents were classified as zoonotic or having zoonotic potential. To determine the zoonotic potential of the diseases, the OIE list for infectious diseases, the “Merck Veterinary Manual” (22), as well as various textbooks (23–26) were consulted. Diseases that are not yet known to have zoonotic potential or are currently being researched have been labeled as “unknown.”

## Results

### Publication Frequency

**Figure 2:**
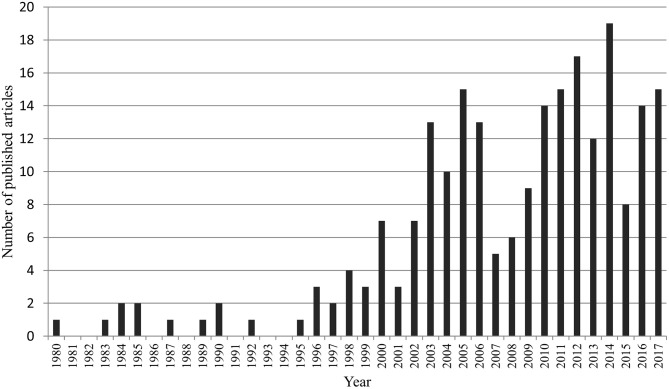
Frequency of publications addressing wildlife diseases in Austria between 1980 and 2017.

**Figure 3:**
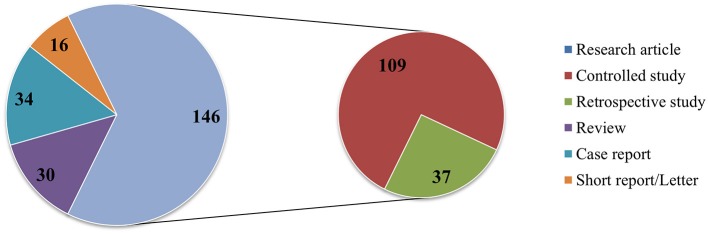
Numerical amount of the different types of scientific publications according to the Springer classification.

After review, we retained 226 publications, published between 1980 and 2017 (Figure 2). With the exception of the years 1981–1982, 1986, 1988, 1991, and 1993–1994, numerous wildlife disease papers were published. From 1995 onwards, there was a continuous increase in wildlife disease publications, with the most articles (*n* = 19) published in 2014. In 2007 and 2015, a drop in publications was noted, followed by an increase in the subsequent year. During the 37 years reviewed, 2000–2017 was most productive period with 89.4% of the retained articles published during this time period. Comparing the average yearly number of publications in the first decade (1980–1989 = 0.8 ± 0.8) to the last decade (2008–2017 = 12.9 ± 4.1) clearly shows a significant increase in interest in wildlife diseases (normality of data was tested by Shapiro-Wilk; two-tailed *t*-test: *p* = 0.01, *t*-statistics = −9.1178). Of the 226 papers, 146 publications were empirical studies, more specifically, 109 controlled studies, and 37 retrospective analyses. The rest was composed of 30 review articles, 34 case reports, and 16 letters or short reports (Figure 3).

### Infectious Agents

**Figure 4:**
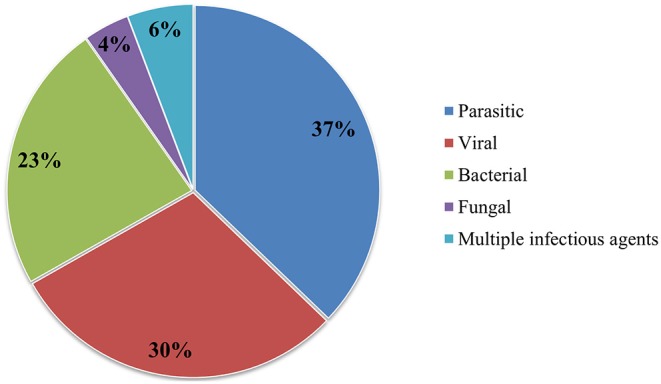
Percentage of the infectious agents mentioned in all publications.

**Figure 5:**
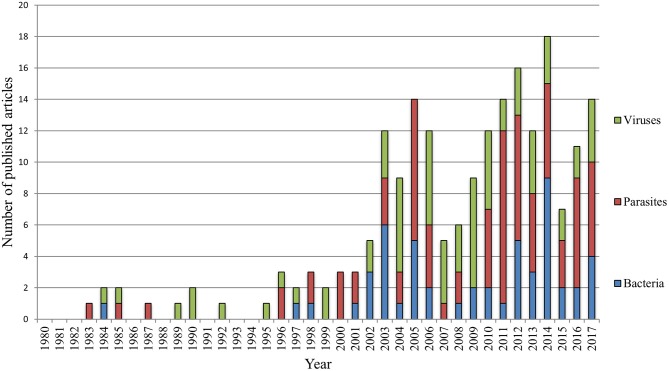
Distribution of publications addressing either bacterial, viral, or parasitic diseases between 1980 and 2017.

**Figure 6:**
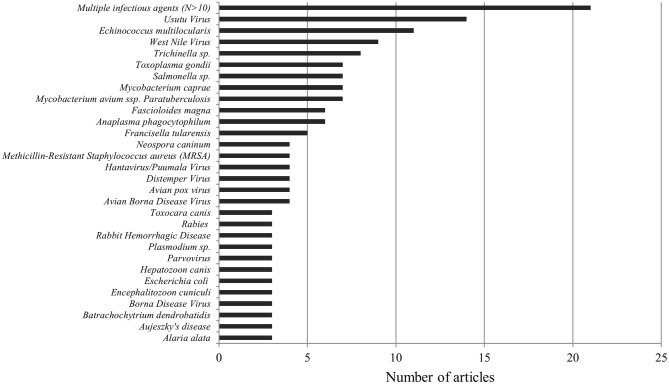
Numerical depiction of diseases mentioned in all publications three times or more.

The largest proportion of publications, namely 84 (37%), addressed infectious diseases caused by parasites (endoparasites: protozoa and helminths, or ectoparasites: arachnids and insects). A total of 67 (30%) of publications discussed viral diseases and 53 (23%) bacterial diseases, 9 (4%) dealt with diseases of fungal origin, and 13 (6%) papers mentioned multiple infectious agents (27–41). We found no studies concerning diseases caused by prions (see Figure 4). In summary, we found 136 pathogens discussed in the 226 publications. For the complete list of all diseases and pathogens found in this study, see Table 1. Of these 136 infectious agents, 84 (62%) were only featured once, 23 (17%) were mentioned twice, and 12 (9%) were discussed three times. The remaining pathogens, namely 17 (12%), were featured more than three times. No clear trend was determined as to changes in which pathogenic agents were reported over the years (see Figure 5). Beyond the publications that discussed multiple pathogens (*N* > 10), Usutu virus was the most studied agent in Austria, with 14 published papers. It was followed by *Echinococcus multilocularis* (*n* = 11), West Nile Virus (*n* = 9), *Trichinella* sp. (*n* = 8), *Toxoplasma gondii, Salmonella* sp., *Mycobacterium caprae*, and *Mycobacterium avium* subsp. *paratuberculosis* with seven publications each. Also noteworthy are *Fascioloides magna* and *Anaplasma phagocytophilum* with six publications each, as well as *Francisella tularensis*, which was mentioned in five papers (see Figure 6).

**Table 1:** Infectious pathogens in alphabetical order, listing their zoonotic potential, notifiable status, animals affected, and authors, as found through our study.

**Disease or pathogen**	**Disease agent**	**Notifiable in Austria**	**OIE notifiable disease**	**Zoonotic potential**	**Affected species**	**References**
Agamid adenovirus 1	Viral	No	No	No	Bearded dragon *(Pogona vitticeps)*	(42)
*Alaria alata*	Parasitic	No	No	Yes	Red fox *(Vulpes vulpes)*, Wild boar *(Sus scrofa)*	(43–45)
*Anaplasma phagocytophilum*	Parasitic	No	No	Yes	Chamois *(Rupicapra rupicapra)*, Ibex *(Capra ibex*), Mouflon *(Ovis gmelini musimon*), Red deer *(Cervus elaphus*), Roe deer *(Capreolus capreolus)*, Timber wolf *(Canis Lupus Occidentalis)*	(46–51)
*Ankylostoma* spp.	Parasitic	No	No	Yes	Cheeta*h (Acinonyx jubatus)*	(52)
*Arboviruses*	Viral	No	No	Yes	Multiple species^*^	(53)
*Ascaris suum*	Parasitic	No	No	Yes	Racoon *(Procyon lotor)*, Red fox *(Vulpes vulpes*), Wild boar *(Sus scrofa)*	(54)
*Ascaridia* spp.	Parasitic	No	No	NA	Northern bald ibis *(Geronticus eremita)*	(55)
*Aspergillus fumigates*	Fungal	No	No	Yes	Falcon *(Falco)*	(56)
*Aspergillus* spp.	Fungal	No	No	Yes	Cheetah *(Acinonyx jubatus)*	(52)
Aujeszky's disease^a^	Viral	Yes	Yes	No	Dog *(Canis lupus familiaris)*, Wild boar *(Sus scrofa)*	(57–59)
Avian Borna disease virus	Viral	No	No	No	Canary bird *(Serinus canaria*) and multiple psittacine species^*^	(60–63)
Avian hepatitis E virus	Viral	No	No	Yes	Common buzzard *(Buteo buteo)*, Feral pigeon *(Columba livia domestica)*, Little owl *(Athene noctua)*, Song thrush *(Turdus philomelos)*	(64)
Avian influenza virus^a^^,^ ^b^	Viral	Yes	Yes	Yes	Coot *(Fulica atra)*, Duck *(Anatidae)*, Egret *(Ardeidae)*, Goose *(Anatidae)*, Grebe *(Podicipedidae*), Swan *(Cygnus)*, and other wild bird species	(65, 66)
Avian pox virus	Viral	No	No	No	Bald eagle *(Haliaeetus leucocephalus)*, Great Bustard *(Otis tarda)*, Great tit *(Parus major)*, and multiple bird species^*^	(67–70)
*Babesia canis*	Parasitic	No	No	No	Eurasian golden jackal *(Canis aureus)*	(71)
*Babesia capreoli*	Parasitic	No	No	Unknown	Chamois *(Rupicapra rupicapra)*, Ibex *(Capra ibex*), Mouflon *(Ovis gmelini musimon*), Red deer *(Cervus elaphus)*, Roe deer *(Capreolus capreolus)*	(48)
*Babesia divergens*	Parasitic	No	Yes	Yes	Chamois *(Rupicapra rupicapra)*, Ibex *(Capra ibex)*, Mouflon *(Ovis gmelini musimon)*, Red deer *(Cervus elaphus)*, Roe deer *(Capreolus capreolus)*	(48, 51)
*Babesia microti*	Parasitic	No	No	Yes	Red fox *(Vulpes vulpes*) and other wild carnivores	(72, 73)
*Babesia vesperuginis*	Parasitic	No	No	Unknown	Bat *(Chiroptera)*	(74)
*Babesia* spp.	Parasitic	No	No	NA	Chamois *(Rupicapra rupicapra)*, Fallow deer *(Dama dama)*, Mouflon *(Ovis gmelini musimon);* Père David's deer *(Elaphurus davidianus)*, Red deer *(Cervus elaphus)*, Roe deer *(Capreolus capreolus)*, Sika deer *(Cervus nippon)*	(75)
*Bartonella* spp.	Bacterial	No	No	Yes	Bank vole *(Clethrionomys glareolus)*, Common vole *(Microtus arvalis)*, Wood mouse *(Apodemus sylvaticus)*, Yellow-necked field mouse *(Apodemus flavicollis)*	(76)
*Batrachochytrium dendrobatidis*	Fungal	No	Yes	Unknown	Alpine newt *(Ichthyosaura alpestris)*, Alpine salamander *(Salamandra atra)*, Crested newt *(Triturus cristatus, T. carnifex, T. dobrogicus)*, Fire and yellow bellied toad *(Bombina bombina, B. variegata)*, Poison dart frogs *(Dendrobatidae)*, Smooth newt *(Lissotriton vulgaris)*, Water frogs *(Pelophylax)*	(77–79)
*Baylisascaris procyonis*	Parasitic	No	No	Yes	Racoon *(Procyon lotor)*, Red fox *(Vulpes vulpes)*, Wild boar *(Sus scrofa)*	(54)
*Baylisascaris* spp.	Parasitic	No	No	Yes	Skunk *(Mephitis mephitis)*	(80)
Borna Disease Virus^c^	Viral	Yes	No	Yes	Bicolored shrew *(Crocidura leucodon*), Cheetah *(Acinonyx jubatus)*, Domestic horse *(Equus ferus caballus)*, and multiple other species^*^	(81–83)
*Borrelia* spp.	Bacterial	No	No	Yes	Bank vole *(Clethrionomys glareolus)*, Common vole *(Microtus arvalis*), Wood mouse *(Apodemus sylvaticus)*, Yellow-necked field mouse *(Apodemus flavicollis)*	(76)
Bot fly larvae “*Cephenemyia auribarbis”*	Parasitic	No	No	Unknown	Red deer *(Cervus elaphus)*	(84)
Bovine viral diarrhea virus^d^	Viral	Yes	Yes	No	Chamois *(Rupicapra rupicapra)*, Fallow deer *(Dama dama)*, Red deer *(Cervus elaphus)*, Roe deer *(Capreolus capreolus)*	(85, 86)
*Brucella microti*	Bacterial	No	No	Unknown	Red fox *(Vulpes vulpes)*	(87, 88)
*Brucella suis*^a^	Bacterial	Yes	Yes	Yes	Red fox *(Vulpes vulpes)*	(89)
*Brucella vulpis*	Bacterial	No	No	Unknown	Red fox *(Vulpes vulpes)*	(90)
*Brucella* spp.^b^	Bacterial	Yes	No	NA	European brown hare *(Lepus europaeus)*, Red fox *(Vulpes vulpes)*	(91, 92)
*Campylobacter* spp.^b^	Bacterial	Yes	No	Yes	Multiple wild bird species^*^	(93, 94)
*Candidatus neoehrlichia* spp.	Bacterial	No	No	Yes	Red fox *(Vulpes vulpes)*	(95)
*Capillaria* spp.	Parasitic	No	No	NA	Northern bald ibis *(Geronticus eremita)*	(55)
*Capillaria boehmi*	Parasitic	No	No	Unknown	Red fox *(Vulpes vulpes)*	(96)
*Capillaria hepatica/Calodium hepaticum*	Parasitic	No	No	Yes	Multiple species^*^	(97–99)
Chelonid Herpesvirus (ChHV)	Viral	No	No	No	Leopard tortoise *(Stigmochelys pardalis)*	(100)
*Chlamydiaceae* spp.	Bacterial	No	No	NA	Multiple wild rodent species^*^	(101)
*Chrysosporium guarroi*	Fungal	No	No	Unknown	Bearded dragon *(Pogona vitticeps)*	(102)
*Clostridium botulinum*^b^*, Avian botulism*	Bacterial	Yes	No	No	Garganey *(Anas querquedula)*, Mallard *(Anas platyrhynchos)*, Northern lapwing *(Vanellus vanellus)* and multiple other wild bird species^*^	(103, 104)
*Clostridium perfringens*	Bacterial	No	No	Yes	Blackbuck *(Antilope cervicapra)*, Collared peccary *(Pecari tajacu)*, Domestic goat *(Capra aegagrus hircus)*, Domestic sheep *(Ovis aries)*, Domestic swine *(Sus scrofa domesticus)*, European reindeer *(Rangifer tarandus)*, Japanese serow *(Capricornis crispus)*, Lechwe *(Kobus leche)*, Waterbuck *(Kobus ellipsiprymnus)*	(105)
*Clostridium septicum*	Bacterial	No	No	Yes	Chamois *(Rupicapra rupicapra)*	(106)
*Coccidia*	Parasitic	No	No	NA	Common hill myna *(Gracula religiosa)*	(107)
*Collyriclum faba*	Parasitic	No	No	Unknown	*S*tarling *(Sturnus vulgaris)*	(108)
*Coxiella burnetii*	Bacterial	No	No	Yes	Bank vole *(Clethrionomys glareolus)*, Common vole *(Microtus arvalis)*, Wood mouse *(Apodemus sylvaticus)*, Yellow-necked field mouse *(Apodemus flavicollis)*	(76)
Crane hepatitis herpesviruses	Viral	No	No	Unknown	Gray crowned crane *(Balearica regulorum)*	(109)
*Cryptococcus* spp.	Fungal	No	No	Yes	Feral pigeon *(Columba livia domestica)*	(110)
*Cryptosporidium* spp.	Parasitic	No	No	Yes	Corn snake *(Pantherophis guttatus)*, Leopard gecko *(Eublepharis macularius)*, and multiple reptile and snake species^*^	(111, 112)
*Cryptosporidium suis*	Parasitic	No	No	Yes	Wild boar *(Sus scrofa)*	(113)
*Cryptosporidium scrofarum*	Parasitic	No	No	Yes	Wild boar *(Sus scrofa)*	(113)
*Demodex* spp.	Parasitic	No	No	No	Cheetah *(Acinonyx jubatus)*	(52)
*Dermatophilus congolensis*	Bacterial	No	No	Yes	Orangutan *(Pongo pygmaeus pygmaeus)*	(114)
*Dermatophytosis (Trichopphyton, Microsporum)*	Fungal	No	No	Yes	Small mammals	(115)
*Devriesea agamarum*	Bacterial	No	No	Unknown	Bearded dragon *(Pogona vitticeps)*	(102)
*Dirofilaria repens*	Parasitic	No	No	Yes	Dog *(Canis lupus familiaris)*, Red fox *(Vulpes vulpes)*	(116)
*Dirofilaria immitis*	Parasitic	No	No	Yes	Dog *(Canis lupus familiaris)*, Red fox *(Vulpes vulpes)*	(116)
Distemper virus	Viral	No	No	No	Badger *(Meles meles)*, Beech marten *(Martes foina)*, Cheetah *(Acinonyx jubatus)*, Dog *(Canis lupus familiaris)*	(82, 117–119)
*Echinococcus multilocularis^b^*	Parasitic	Yes	Yes	Yes	Beaver *(Castor fiber)*, Dog *(Canis lupus familiaris)*, Domestic cat *(Felis silvestris catus)*, Red fox *(Vulpes vulpes)*, and multiple mammalian or rodent species^*^	(54, 99, 120–128)
*Eimeria leporis*	Parasitic	No	No	No	European brown hare *(Lepus europaeus)*	(123)
*Encephalitozoon cuniculi*	Parasitic	No	No	Yes	Bearded dragon *(Pogona vitticeps)*, Common vole *(Microtus arvalis)*, European brown hare *(Lepus europaeus)*, European water vole *(Arvicola terrestris)*	(129–131)
*Encephalitozoon* spp.	Parasitic	No	No	Yes	Multiple species^*^	(132)
Encephalomyocarditis virus	Viral	No	No	Yes	Domestic cat *(Felis silvestris catus)* and wild rodents	(133)
*Entamoeba invadens*	Parasitic	No	No	No	Boa constrictor *(Boa constrictor)*	(134)
*Entamoeba* spp.	Parasitic	No	No	NA	Multiple snake species^*^	(112)
ESBL/AmpC producing Enterobacteriaceae	Bacterial	No	No	Yes	Rook *(Corvus frugilegus)*	(135)
*Escherichia coli*	Bacterial	No	No	Yes	Mouflon *(Ovis gmelini musimon)* and multiple wild bird species^*^	(93, 94, 136)
European brown hare *(Lepus europaeus)* syndrome	Viral	No	No	No	European brown hare *(Lepus europaeus)*, Rabbit *(Oryctolagus cuniculus)*	(137, 138)
*Fascioloides magna*	Parasitic	No	No	No	Fallow deer *(Dama dama)*, Red deer *(Cervus elaphus)*, Roe deer *(Capreolus capreolus)*, and otherwild ungulates and ruminants	(139–144)
Feline Herpes Virus 1	Viral	No	No	No	Cheetah *(Acinonyx jubatus)*	(82, 145)
Flavivirus, mosquito-borne	Viral	No	No	Yes	Multiple species^*^	(146)
*Francisella tularensis^b^*	Bacterial	Yes	Yes	Yes	Dog *(Canis lupus familiaris)*, European brown hare *(Lepus europaeus)*, Red fox *(Vulpes vulpes)*	(18, 89, 91, 147, 148)
*Fur mites (Lynxacarus mustelae)*	Parasitic	No	No	Unknown	Beech marten *(Martes foina)*	(149)
*Geopetitia aspiculata*	Parasitic	No	No	Unknown	Bearded barbet *(Lybius dubius)*, Black-faced dacnis *(Dacnis lineata)*, Brown-breasted barbet *(Lybius melanopterus)*, Sociable weaver *(Philetairus socius)*, Steere's liocichla *(Liocichla steerii)*	(150)
Hantavirus/Puumala Virus^b^	Viral	Yes	No	Yes	Bank vole *(Clethrionomys glareolus)*, Common vole *(Microtus arvalis)*, Domestic cat *(Felis silvestris catus*), House mouse *(Mus musculus)*, Wood mouse *(Apodemus sylvaticus)*, Yellow-necked field mouse *(Apodemus flavicollis)*, and other wild rodents	(76, 133, 151, 152)
*Hepatozoon canis*	Parasitic	No	No	No	Eurasian golden jackal *(Canis aureus)*, Red fox *(Vulpes vulpes)*	(71, 72, 153)
*Herpesvirus*	Viral	No	No	NA	Tortoises *(Testudinidae)*	(154)
*Histoplasma capsulatum* var. *Capsulatum*	Fungal	No	No	Yes	Badger *(Meles meles)*	(155)
*Iridovirus (*Genus *Ranavirus)*	Viral	No	No	Unknown	Leopard tortoise *(Stigmochelys pardalis)*	(100)
*Klebsiella pneumoniae*	Bacterial	No	No	Yes	Mouflon *(Ovis gmelini musimon)*	(136)
Koala retrovirus (KoRV)	Viral	No	No	Unknown	Koala *(Phascolarctos cinereus)*	(156)
*Leishmania infantum*	Parasitic	No	Yes	Yes	Eurasian golden jackal *(Canis aureus)*	(71)
*Leptospira interrogans*^b^	Bacterial	Yes	No	Yes	European brown hare *(Lepus europaeus)*, Wild boar *(Sus scrofa)*	(91, 157)
*Leptospira* spp.	Bacterial	No	No	Yes	Bank vole *(Clethrionomys glareolus*), Common vole *(Microtus arvalis)*, Wood mouse *(Apodemus sylvaticus)*, Yellow-necked field mouse *(Apodemus flavicollis)*	(76)
*Listeria monocytogenes*^b^	Bacterial	Yes	No	Yes	Water fowl *(Anatidae)*, Red deer *(Cervus elaphus)*, Roe deer *(Capreolus capreolus*), Chamois *(Rupicapra rupicapra)*, Wild boar *(Sus scrofa)*, Mouflon *(Ovis gmelini musimon)*, and other wild and domestic ruminants	(158, 159)
*Listeria* spp.	Bacterial	No	No	NA	Water fowl *(Anatidae*) and wild and domestic ruminants	(159)
Lymphocytic choriomeningitis virus	Viral	No	No	Yes	Bank vole *(Clethrionomys glareolus)*, Common vole *(Microtus arvalis)*, Wood mouse *(Apodemus sylvaticus)*, Yellow-necked field mouse *(Apodemus flavicollis)*	(76)
Malignant catarrhal fever virus	Viral	No	No	No	Chamois *(Rupicapra rupicapra)*, Fallow deer *(Dama dama)*, Red deer *(Cervus elaphus*), Roe deer *(Capreolus capreolus)*	(160)
Methicillin-resistant *Staphylococcus aureus* (MRSA)	Bacterial	No	No	Yes	Eurasian Lynx *(Lynx lynx*), European brown hare *(Lepus europaeus)*, Hedgehog *(Erinaceus europaeus)*, Otter *(Lutra lutra)*, Rook *(Corvus frugilegus)*	(93, 135, 161, 162)
*Monocercomonas* spp.	Parasitic	No	No	Unknown	Multiple snake species^*^	(112)
*Mycobacterium avium* ssp. *Paratuberculosis*^e^	Bacterial	Yes	Yes	Yes	Capercaillie *(Tetrao urogallus)*, Cattle *(Bos taurus)*, Chamois *(Rupicapra rupicapra*), European brown hare *(Lepus europaeus)*, Fallow deer *(Dama dama)*, Ibex *(Capra ibex)*, Mouflon *(Ovis gmelini musimon)*, Mountain hare *(Lepus timidus)*, Red deer *(Cervus elaphus)*, Roe deer *(Capreolus capreolus)*, Red fox *(Vulpes vulpes)*, Yellow-necked field mouse *(Apodemus flavicollis)*	(163–169)
*Mycobacterium avium* subsp. *silvaticum*	Bacterial	No	Yes	Yes	Ural owl *(Strix uralensis)*	(170)
*Mycobacterium bovis*^a^^,^ ^b^	Bacterial	Yes	Yes	Yes	Badger *(Meles meles)*, Red deer *(Cervus elaphus)*, Wild boar *(Sus scrofa)*	(171)
*Mycobacterium caprae*^b^	Bacterial	Yes	Yes	Yes	Camel *(Camelus)*, Cattle *(Bos taurus)*, Domestic goat *(Capra aegagrus hircus)*, Red deer *(Cervus elaphus)*, Wild boar *(Sus scrofa)*	(16, 172–177)
*Mycoplasma conjunctivae*	Bacterial	No	No	Yes	Chamois *(Rupicapra rupicapra)*, Domestic goat *(Capra aegagrus hircus)*, Domestic sheep *(Ovis aries)*, Domestic swine *(Sus scrofa domesticus)*, Ibex *(Capra ibex)*	(178, 179)
*Myxozoa*	Parasitic	No	No	NA	Mole *(Talpa europaea)*	(180)
*Neospora caninum*	Parasitic	No	No	No	Common vole *(Microtus arvalis)*, Dog *(Canis lupus familiaris)*, European brown hare *(Lepus europaeus)*, European water vole *(Arvicola terrestris)*, Red fox *(Vulpes vulpes)*, Parma wallaby *(Macropus parma)*	(129, 181–183)
Orthopoxvirus	Viral	No	No	Yes	Domestic cat *(Felis silvestris catus)* and wild rodents	(133)
Parapoxvirus	Viral	No	No	Yes	Domestic cat *(Felis silvestris catus*) and wild rodents	(133)
Parvovirus	Viral	No	No	No	Badger *(Meles meles)*, Cheetah *(Acinonyx jubatus)*	(52, 82, 118)
*Pasteurella* spp.	Bacterial	No	No	NA	Chamois *(Rupicapra rupicapra)*	(184)
*Pharyngomyia picta*	Parasitic	No	No	Unknown	Red deer *(Cervus elaphus)*	(84)
*Plasmodium* spp.^b^	Parasitic	Yes	No	No	Humboldt penguins *(Spheniscus humboldti)*, King penguins *(Aptenodytes patagonicus)*, Puffins *(Fratercula arctica)*, Rockhopper penguins *(Eudyptes chrysocome)*, and multiple wild bird species^*^	(185–187)
Psittacine beak and feather disease virus	Viral	No	No	Unknown	Budgerigar *(Melopsittacus undulatus)*	(188)
Rabies^a^^,^ ^b^	Viral	Yes	Yes	Yes	Red fox *(Vulpes vulpes)*	(189–191)
Rabbit hemorrhagic disease	Viral	No	Yes	No	European brown hare *(Lepus europaeus)*, Rabbit *(Oryctolagus cuniculus)*	(137, 138, 192)
Rat hepatitis E virus	Viral	No	No	Yes	Black rat *(Rattus rattus)*, Norway rat *(Rattus norvegicus)*	(193)
*Rickettsia* spp.	Bacterial	No	No	Yes	Bank vole *(Clethrionomys glareolus)*, Common vole *(Microtus arvalis)*, Wood mouse *(Apodemus sylvaticus)*, Yellow-necked field mouse *(Apodemus flavicollis)*	(76)
Roe deer *(Capreolus capreolus)* papillomavirus	Viral	No	No	No	Red deer *(Cervus elaphus)*	(194)
*Salmonella enterica^b^*	Bacterial	Yes	No	Yes	Red fox *(Vulpes vulpes)* and multiple reptile species^*^	(195, 196)
*Salmonella* spp.^b^	Bacterial	Yes	No	NA	Chamois *(Rupicapra rupicapra)*, Lizards *(Squamata)*, Mouflon *(Ovis gmelini musimon)*, Red deer *(Cervus elaphus)*, Roe deer *(Capreolus capreolus)*, Snakes *(Serpentes)*, Tortoises *(Testudinidae)*, Turtles *(Chelonii)*, Wild boar *(Sus scrofa)*, and multiple wild bird, amphibian, reptile and other species^*^	(93, 94, 158, 197–200)
Scabies/Sarcoptic mange^a^	Parasitic	Yes	No	Yes	Chamois *(Rupicapra rupicapra)*, Ibex *(Capra ibex)*	(201, 202)
*Staphylococcus aureus*	Bacterial	No	No	Yes	Multiple species^*^	(203)
*Syngamus trachea*	Parasitic	No	No	Yes	Northern bald ibis *(Geronticus eremita)*	(55)
*Taenia crassiceps*	Parasitic	No	No	Yes	Common vole *(Microtus arvalis)*, European water vole *(Arvicola terrestris)*	(99, 204)
*Taenia taeniaeformis*	Parasitic	No	No	Yes	Common vole *(Microtus arvalis)*, European water vole *(Arvicola terrestris)*	(99, 204)
*Taenia* spp.	Parasitic	No	No	Yes	Cheetah *(Acinonyx jubatus)*	(52)
*Theileria* spp.	Parasitic	No	Yes	No	Chamois *(Rupicapra rupicapra)*, Fallow deer *(Dama dama)*, Mouflon *(Ovis gmelini musimon)*, Père David's deer *(Elaphurus davidianus)*, Red deer *(Cervus elaphus)*, Roe deer *(Capreolus capreolus)*, Sika deer *(Cervus nippon)*	(75, 205)
Tickborne encephalitis virus^b^ (TBEV)	Viral	Yes	No	Yes	Mouflon *(Ovis gmelini musimon)*, Roe deer *(Capreolus capreolus)*	(206, 207)
*Toxascaris leonina*	Parasitic	No	No	No	Cheetah *(Acinonyx jubatus)*	(52)
*Toxocara canis*		No	No	Yes	Dog *(Canis lupus familiaris)*, Domestic cat *(Felis silvestris catus)*, Racoon *(Procyon lotor)*, Red fox *(Vulpes vulpes)*, Wild boar *(Sus scrofa)*	(54, 208, 209)
*Toxocara cati*	Parasitic	No	No	Yes	Dog *(Canis lupus familiaris)*, Domestic cat *(Felis silvestris catus)*, Red fox *(Vulpes vulpes)*	(208, 209)
*Toxocara mystax*	Parasitic	No	No	Yes	Cheetah *(Acinonyx jubatus)*	(52)
*Toxoplasma gondii*	Parasitic	No	No	Yes	Bank vole *(Clethrionomys glareolus)*, Common vole *(Microtus arvalis)*, European water vole *(Arvicola terrestris)*, Pallas cat *(Otocolobus manul)*, Racoon *(Procyon lotor)*, Red fox *(Vulpes vulpes)*, Wild boar *(Sus scrofa)*, Wood mouse *(Apodemus sylvaticus)*, Yellow-necked field mouse *(Apodemus flavicollis)* and multiple other species^*^	(54, 76, 99, 181, 182, 210, 211)
*Treponema* spp.	Bacterial	No	No	Unknown	European brown hare *(Lepus europaeus)*	(212)
*Trichinella britovi*	Parasitic	No	Yes	Yes	Red fox *(Vulpes vulpes)*	(213)
*Trichinella pseudospiralis*	Parasitic	No	Yes	Yes	Wild boar *(Sus scrofa)*	(214)
*Trichinella* spp.	Parasitic	No	Yes	Yes	Domestic swine *(Sus scrofa domesticus)*, Racoon *(Procyon lotor)*, Red fox *(Vulpes vulpes)*, Wild boar *(Sus scrofa)*	(54, 215–221)
*Trichomonas gallinae*	Parasitic	No	No	No	Bearded vulture *(Gypaetus barbatus)*, Budgerigar *(Melopsittacus undulatus)*, Canary bird *(Serinus canaria)*, Eurasian collared dove *(Streptopelia decaocto)*, European greenfinch *(Chloris chloris)*, Feral pigeon *(Columba livia domestica)*, Hawfinch *(Coccothraustes coccothraustes)*, Racing pigeons *(Columba livia domestica)*, Yellowhammer *(Emberiza citrinella)*	(222, 223)
*Trichophyton terrestre*	Fungal	No	No	Unknown	Madagascar day gecko *(Phelsuma m. madagascariensis)*	(224)
*Trichostrongylus tenius*	Parasitic	No	No	Yes	Northern bald ibis *(Geronticus eremita)*	(55)
*Tritrichomonas fetus*	Parasitic	No	No	No	Quail *(Coturnix coturnix)*	(225)
Usutu Virus	Viral	No	No	Yes	Black bird *(Turdus merula)*, Blue tit *(Cyanistes caeruleus)*, Egyptian vulture *(Neophron percnopterus)*, Eurasian eagle owl *(Bubo bubo)*, Great gray owl *(Strix nebulosa)*, House sparrow *(Passer domesticus)*, Owls *(Strigiformes)*, Snowy owl *(Bubo scandiacus)*, Swallow *(Hirundinidae)*, White stork *(Ciconia ciconia)*, Ural owl *(Strix uralensis)*, and multiple wild bird and other species^*^	(226–239)
Verocytotoxin-producing *Escherichia coli*^b^	Bacterial	Yes	No	Yes	Chamois *(Rupicapra rupicapra)*, Cattle *(Bos taurus)*	(240)
West Nile Virus^b^^,^ ^c^	Viral	Yes	Yes	Yes	Bearded vulture *(Gypaetus barbatus)*, Domestic horse *(Equus ferus caballus)*, Goshawk *(Accipiter gentilis)*, Gyrfalcon *(Falco rusticolus)*, Kea *(Nestor notabilis)*, Snowy owl *(Bubo scandiacus)*, and multiple other bird species^*^	(237, 241–248)
*Yersinia pseudotuberculosis*	Bacterial	No	No	Yes	Cheetah *(Acinonyx jubatus)*	(52)

a *Notifiable diseases according to the § 16. Federal law on epizootics in Austria*.

b *Notifiable diseases according to the Tuberculosis Act, Epidemic Act 1950, and Sexually Transmitted Diseases Act in Austria*.

c *Notifiable disease in Austria, as part of the Equine encephalitis disease complex*.

d *Notifiable according to the BVD Regulation 2007, BGBl. II No. 178/2007*.

e Notifiable according to the Paratuberculosis Ordinance, BGBl. II No. 48/2006^*^.

* *Multiple species/other species, indicates more than 10 (N > 10) mentioned animal species*.

### Zoonotic Potential

**Figure 7:**
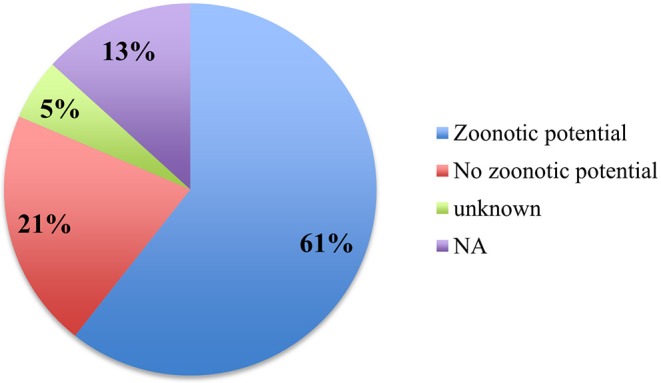
Percentage of publications discussing the zoonotic potential of diseases.

**Figure 8:**
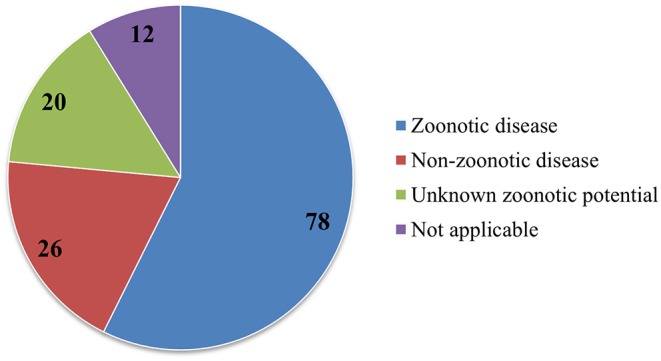
Numbers of diseases with or without zoonotic potential, as well as diseases with currently unknown zoonotic potential.

The review shows that 61% of papers discussed pathogens that have zoonotic potential, 21% of papers discussed diseases without zoonotic potential, and 5% discussed diseases with unclear zoonotic potential that would need further research (see Figures 7, 8).

### Notifiable Status

**Figure 9:**
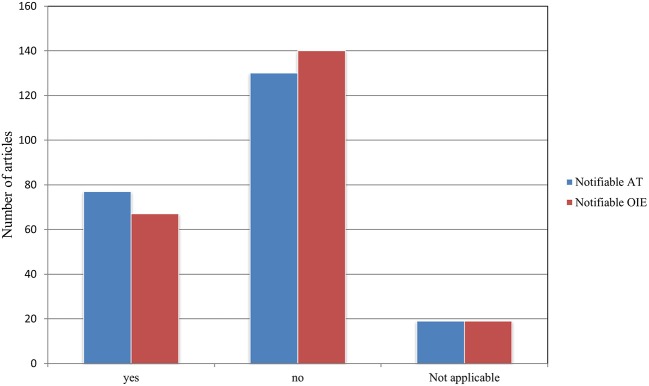
Number of publications addressing diseases which are notifiable according to Austrian legislation and/or the OIE–World Organization for Animal Health compared to those addressing non-notifiable diseases.

**Figure 10:**
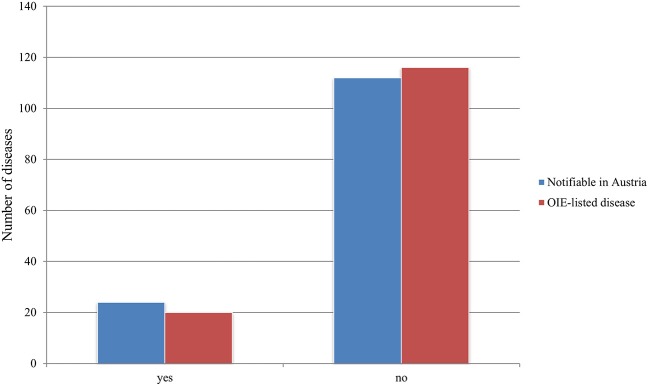
Number of notifiable diseases described in retained papers according to Austrian legislation and the OIE compared to non-notifiable diseases.

A total of 77 publications addressed diseases notifiable in Austria according to Austrian legislation including the § 16. Federal law on epizootics, the Tuberculosis Act (1968), the Epidemic Act (1950), the Sexually Transmitted Diseases Act (1945), the BVD Regulation (2007), and the Paratuberculosis Ordinance (2006), while 67 publications mentioned OIE notifiable diseases (See Supplementary Tables 1, 2). Of the 77 publications addressing notifiable diseases in Austria, 48 also addressed notifiable diseases according to the OIE list. For 19 publications, our criteria were not applicable, as they did not mention an exact pathogen but multiple pathogens (*N* > 10) (see Figure 9). Of the 136 diseases detected, 24 diseases are notifiable according to current Austrian law. Six of these 24 diseases are notifiable as per the Austrian § 16. Federal law on epizootics, the remaining diseases are notifiable according to the above-mentioned laws (See Supplementary Tables 1, 2). Moreover, 20 diseases were found to be OIE-listed diseases (see Figure 10). Lastly, of the 24 diseases notifiable in Austria, 11 are also notifiable according to the OIE list.

### Prevalence and Confidence Interval

**Figure 11:**
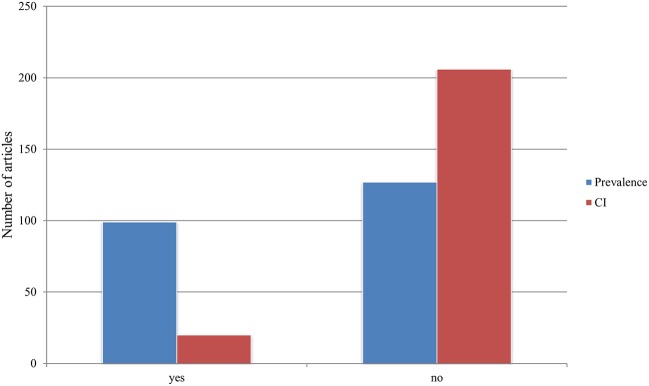
Representation of the specification of prevalence and confidence interval (CI) in all the publications.

Prevalence was indicated in a total of 99 (44%) publications. The confidence intervals, on the other hand, were only mentioned in 20 (9%) publications (see Figure 11).

### Animal Species

**Figure 12:**
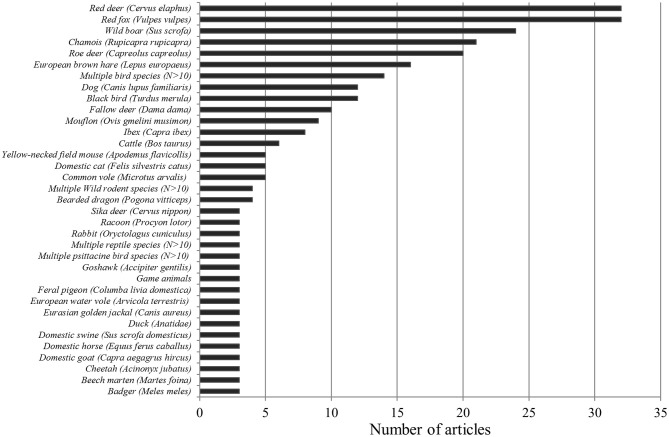
Numerical occurrence of the individual wild animal species or group in the investigated literature.

Both red deer (*Cervus elaphus*) and red foxes (*V. vulpes*) were each mentioned in 32 papers. These numbers were followed by wild boar (*S. scrofa*) (*n* = 25), chamois (*Rupicapra rupicapra*) (*n* = 21), roe deer (*Capreolus capreolus*) (*n* = 20), European brown hare (*Lepus europeus*) (*n* = 16), blackbird (*Turdus merula*) (*n* = 12), and fallow deer (*Dama dama*) (*n* = 10). Among the 10 most frequently described animal species, the domestic dog (*Canis lupus familiari*s) was the only domestic animal species. It occurred in a total of 12 papers. Furthermore, in 14 papers, multiple bird species were listed (see Figure 12).

In summary, we found 131 animal species or groups (e.g., genera, families, and orders) in the 226 publications. Of these, 58 were birds, followed by 55 mammal species, and 2 marsupial species. Reptiles and amphibians appear underrepresented by nine species or groups and seven species, respectively. Thirteen more categories were formed to describe those papers addressing more than 10 species or not stating an exact species. These included multiple bird, rodent, reptile psittacine bird, snake, amphibian and mammalian species, and domestic ruminants, wild ruminants, wild carnivores, wild ungulates, and game animals. Ninety six species or categories were mentioned once, whereas 12 were mentioned twice.

### Risk Group Interfaces

**Figure 13:**
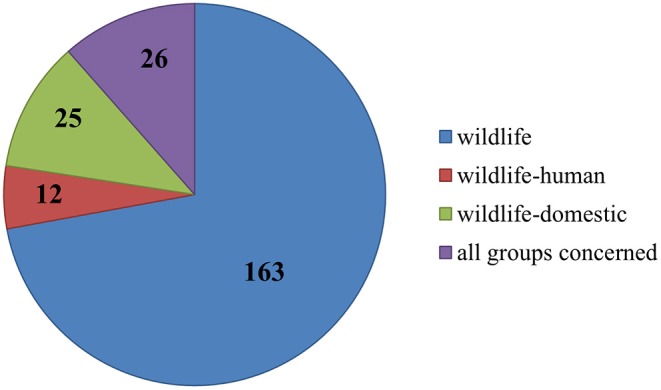
Numerical allocation of the observed interfaces addressed in all publications.

A total of 163 publications addressed diseases that occurred exclusively in wild animals, conducted research solely with wild animals or took samples only from wildlife. In contrast, 12 papers dealt with diseases at the wildlife-human interface, and all of these diseases were zoonotic. In these 12 studies, humans and animals were sampled or results reported in humans and animals were discussed. Moreover, 25 publications addressed transmission and prevalence of diseases at the wildlife-livestock interface. Last but not least, 26 publications pertained to all interfaces, i.e., between wild animals, domesticated animals, and humans (see Figure 13).

### Distinct Study Features

**Figure 14:**
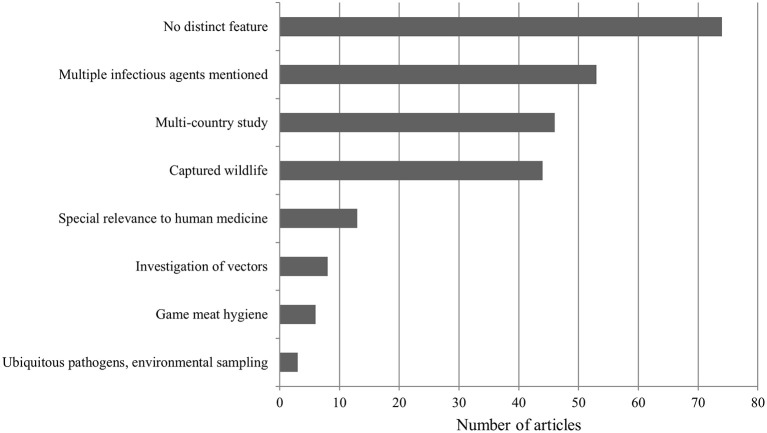
Distribution of different distinct features exhibited by all publications.

A total of 74 papers did not exhibit a specific trait according to our classification and were categorized under “No distinct feature.” In 53 publications, more than one specific pathogenic agent was mentioned. Furthermore, 46 papers referenced countries other than Austria. Diseases that emerged in captive wildlife were discussed in 44 publications. Thirteen studies addressed diseases emerging in wildlife but centered on human medicine (Figure 14). These included sampling of specific at-risk groups (e.g., hunters, veterinarians, animal keepers). Moreover, six papers dealt with game meat hygiene, and lastly, three publications were about environmental sampling for ubiquitous pathogens (e.g., *Clostridium botulinum*, avian botulism).

## Discussion

According to the WHO, 75% of new EIDs or emerging diseases originate from animal reservoirs and thus have zoonotic potential. In the last 30 years, these EIDs have appeared as a consequence of the intensifying codependence in multiuse landscapes at the interface between animals and humans (249). This review provides a first insight into the state of knowledge on wildlife diseases in Austria between 1980 and 2017.

This literature review identified 226 publications between 1980 and 2017. More than half of the publications (65%) involved actively conducted research. The review also revealed that 15% of all papers were case reports. A majority of these case reports were based on pathology reports from the NRL of the AGES, the Institute of Pathology and the Research Institute of Wildlife Ecology at the University of Veterinary Medicine in Vienna. The existence of several facilities addressing wildlife pathogens and disease appears to have had a positive impact on the generation of knowledge in this field.

In comparison to a similar study from the Republic of Korea, which focused on the time span between 1982 and 2014 and found an average rate of 6.1 publications per year, our review yielded a higher total number of publications as well as a higher average amount of publications per year. (1). However, it has to be mentioned that our study covered a longer period of time. In the Korean review, viral diseases were reported on more often than in Austria. The most frequently researched diseases in Korea were avian influenza virus and rabies. In Austria, Usutu virus and *E. multilocularis* clearly dominated. As far as interfaces are concerned, research in Korea focused primarily on human and livestock diseases, whereas in Austria the main focus was on diseases in wildlife. In Korea, 24 zoonotic diseases were reported, whereas in Austria 78 have been investigated since 1980. Fifteen diseases that are notifiable according to the OIE list were mentioned in the Korean review; in contrast, 20 were reported in Austria. Birds dominated in the Korean review, while mammals, namely the red fox (*V. vulpes*) and the red deer (*C. elaphus*), were most reported on in Austria (1).

### Infectious Agents

Parasitic diseases accounted for 37% of the papers, 30% were viral, and 23% were bacterial. Fungal diseases were only mentioned in 4% of all studies. Furthermore, we did not find a single study on transmissible spongiform encephalopathies or other diseases caused by prions. This may be due to the fact that only eight cases of BSE were detected in Austria between 2001 and 2010 (250). Austria was also declared as a country with negligible BSE risk by the OIE in 2012. However, it is prudent not to ignore TSEs in wildlife in view of the sudden emergence of chronic wasting disease in reindeer (*Rangifer tarandus*) and moose (*Alces alces*) in Finland and Norway in April 2016 (251).

Viral diseases were addressed in 30% of publications. The most studied viral pathogens were Usutu Virus and West Nile Virus. Since its first detection in 2001, Usutu Virus has been responsible for massive die-off of blackbirds (*T. merula*) in Europe. The first two human cases of Usutu Virus were detected in 2003 in Austria, generating a greater interest in this vector-borne disease (252). To a much lesser extent, avian Borna disease virus, avian pox virus, distemper virus, and Hantavirus/Puumala virus were also investigated.

Parasitic diseases accounted for 37% of papers. *E. multilocularis, Trichinella* sp., *T. gondii, F. magna, A. phagocytophilum*, and *Neospora caninum* were most often reported on. With the exception of *F. magna* and *N. caninum*, all of these are zoonotic.

When it comes to the bacterial pathogens, the most reported bacteria were *Salmonella* sp., *M. caprae* and *M. avium* subsp. *paratuberculosis, F. tularensis*, and methicillin-resistant *Staphylococcus aureus*. These are all zoonotic, potentially food borne and thus of high relevance to human health. The risk of a spillover or spillback of these bacterial diseases from wildlife to livestock is potentially quite high, as livestock and wildlife share mountain pastures in Austria.

*Batrachochytrium dendrobatidis*, a fungus that infects amphibians and is responsible for the global decline and even extinction of amphibians, was the most reported fungi and a classic EIDs (253).

### Zoonotic Potential

The recovered papers are biased toward zoonotic diseases (61% *n* = 78). This is most likely due to funding being more readily available for diseases representing a human health risk. However, wildlife diseases without zoonotic potential should not be ignored, as they can also pose a risk to society, for example by causing major direct economic losses (e.g., ASF) and the loss of ecosystem services through species loss.

### Notifiable Status

Of the 35 notifiable animal diseases in the Austrian federal law on epizootics, 7 were reported in the examined literature. In addition, the BVD Regulation 2007, BGBl. II No. 178/2007, and the Paratuberculosis Ordinance, BGBl. II No. 48/2006^*^, were of relevance, as both BVD and paratuberculosis were reported in wildlife (See Supplementary Tables 1, 2). The OIE list includes 117 diseases that can possibly infect or infest animals (254). In this review, we noted 20 diseases that are notifiable according to the OIE. Of the 29 diseases discussed more than two times, seven are notifiable according to the OIE list and the Austrian legislation, four are only notifiable in Austria, and three are only notifiable according to the OIE list.

### Prevalence and Confidence Interval

Reporting prevalence is essential and provides insight into the epidemiological situation of a pathogen or disease entity in the sampled region. The disease prevalence was stated in 44% of publications. Of concern is that CI were only mentioned in 9% of the papers, thereby seriously limiting the significance of most studies and respective results. We found that 17 publications that provided a confidence interval for their results were research articles (e.g., controlled studies or retrospective studies). Providing confidence intervals did not increase over the 37 years reviewed. The authors of this study are of the opinion that appropriate descriptive statistics are not sufficiently employed in Austrian wildlife disease research at the present time.

### Animal Species

Of roughly 626 different vertebrate species (including fish) native to Austria, only 21% have been addressed in this review. This demonstrates a huge untapped research potential in wildlife health research.

#### Mammals

The most studied mammalian species were the red fox (*V. vulpes*), the red deer (*C. elaphus*), the wild boar (*S. scrofa*), the chamois (*R. rupicapra*), the roe deer (*C. capreolus*), and the European brown hare (*L. europeus*). It is worth mentioning that these species represent the main game species in Austria. Again, it becomes clear that the primary focus of research has lain on species that serve as food source and offer recreational opportunities for humans. These animals pose a higher risk of transmitting zoonoses, generate funding, and therefore are more interesting to research. The wild boar is a species that has been of great interest to research, as populations are rapidly expanding both in absolute numbers and spatial distribution. Similar to many European countries, wild boar population management is increasingly difficult in Austria. One reason for the rapid reproduction of wild boar is climate change, resulting in increased food availability from beech nuts and acorns coupled with an increased number of litters and offspring survival. Since infection pressure potentially increases with population density, the wild boar population poses an important potential risk for domesticated pigs and humans from ASF or *Trichinella* spp. (214, 255).

#### Birds

The most studied bird species in Austria was the blackbird (*T. merula*) with references in 12 publications. Interestingly, all studies about blackbirds focused on Usutu virus related to the outbreak in Europe in 2000–2001. The mosquito-borne zoonotic Usutu virus has caused widespread deaths in blackbirds. Before the emergence of the disease in Austria, no previous research on this virus existed. Besides blackbirds, goshawks (*Accipiter gentilis*), feral pigeons (*Columba livia domestica*), and ducks were also of special interest to research. The Usutu virus highlights the fact that a large proportion of research related to wildlife diseases is opportunistic and initially reactive.

#### Reptiles and Amphibians

The captive bearded dragon (*Pogona vitticeps*) was the most studied reptile in Austria between 1980 and 2017 and appeared in four studies. However, no disease was mentioned more than once. Moreover, we were not able to identify any individual amphibian species that appeared more than once in the investigated publications. The reason for this low number of studies could be the scarcity of amphibian and reptile species in Austria. There are only 21 known native amphibian species and 16 reptile species in Austria (10).

### Risk Group Interfaces

The majority (72%) of publications either prospectively sampled wild animal species or evaluated research results in retrospective studies. Numerous publications address the possibility of transmission of pathogens between the different interfaces (e.g., wildlife-livestock, wildlife-human). The remaining studies (28%) dealt with diseases affecting the interfaces between wildlife, livestock, and humans. These papers actively collected samples or performed retrospective studies on existing data across different interfaces. Our study shows that most of the publications addressed the fact that wildlife diseases can emerge in and cross various interfaces.

### Distinct Study Features

We were able to identify seven categories within which we could further classify a subset of the papers. Publications could also exhibit more than one distinct feature. Fifty-three publications addressed more than one pathogen of which 42 were research papers. Six publications represented reviews. Furthermore, in 46 studies, countries other than Austria were mentioned. Only 44 studies dealt with captive wildlife or exotic pets. Research at the wildlife-human interface was documented in 13 studies, and these sampled both animals and humans. As the interface between wildlife and humans expands across Austria, it appears prudent to increase research and facilitate human and veterinary medical collaborations. Disease research on trichinosis, tularemia, tuberculosis, and echinococcosis would especially benefit from such collaborative one-health approaches.

## Conclusion

Wildlife diseases will challenge research and medicine alike for years to come, as major changes due to the growing human population and environmental impacts increase. These impacts contribute to increased occurrence of diseases originating in wild animal species spilling over to humans. This review shows that increasing numbers of studies are being conducted in Austria. However, there is still room for further research. With the exception of prions, all infectious agents (viruses, bacteria, parasites, and fungi) have been investigated. Although there have been numerous studies on zoonoses, we have to stress the importance for future research to focus more strongly on disease dynamics and transmission at the wildlife-human interface. We acknowledge that wildlife disease studies frequently lack prevalence data, or data are of poor quality, to perform robust prevalence calculations. However, it appears that data essential to prevalence statement are not always available, especially in single case reports or due to the bias produced by passive surveillance sampling. Therefore, researchers may often decide not to include prevalence data, since it could be misinterpreted or even speculative. We would suggest that future wildlife disease-related studies should include prevalence data if possible, or state other appropriate measures and methods for statistical and epidemiological characterization of a disease event. Similarly, it appears important to expand the scope of the species investigated in the future. Funding is most often only available during impactful events, such as outbreaks, but is notably lacking for baseline research on wildlife, ecosystems, and diseases. Our assumption that there would be an increase in the number of publications on wildlife diseases in Austria over the period examined was confirmed. Considering our hypothesis, spatiotemporal indicators would have been beneficial to our review. However, due to the quality of the available data, its variability in consistency regarding study design, objectives, and methodologies of the investigated studies (e.g., multiple sampling sites, sampling across longer periods of time, multiple diseases in the same study), stating spatiotemporal indicators would have exceeded the clearly defined scope of this initial study. We believe this question would be best addressed in a separate and future analysis. We hope that this review provides information about the current research status for wildlife ecologists, veterinarians, and officials alike and incentivizes further research that employs “One Health” approaches.

## Author Contributions

NT did the literature research and wrote the manuscript. CW planned the review concept and working steps, and edited the manuscript.

### Conflict of Interest

The authors declare that the research was conducted in the absence of any commercial or financial relationships that could be construed as a potential conflict of interest.

## Abbreviations

ADNS: Animal Disease Notification System
AGES: Austrian Agency for Health and Food Safety
ASF: African swine fever
AT: Austria
AVR: Annual Veterinary Report
BSE: Bovine spongiform encephalopathy
BVD: Bovine viral diarrhea
CI: Confidence interval
CWD: Chronic wasting disease
EFSA: European Food Safety Authority
EID: Emerging infectious disease
EU: European Union
KVG: Kommunikationsplattform VerbraucherInnengesundheit
MRSA: *Methicillin-resistant Staphylococcus aureus*
NA: Not applicable
NCBI: National Center for Biotechnology Information
NRL: National Reference Laboratory of the AGES
OIE: World Organization for Animal Health
RZZA: Report of Zoonoses and Zoonotic Agents
TSE: Transmissible spongiform encephalopathy
TSG, Tierseuchengesetz: Federal law on epizootics
WNV: West Nile Virus.
