# Supplementary material for: Infectious Wildlife Diseases in Austria—A Literature Review From 1980 Until 2017
Source: Front Vet Sci. 2020 Feb 21;7:3. doi: 10.3389/fvets.2020.00003 (PMC7046627; doi:10.3389/fvets.2020.00003)
Supplement: Supplementary file 1 [file Table_1.docx]

Supplementary Material

Infectious wildlife diseases in Austria - A literature review from 1980 until 2017

**Nina Eva Trimmel^1^*, Chris Walzer^1, 2^**

^1^ Department of Integrative Biology and Evolution University of Veterinary Medicine Vienna, Research Institute of Wildlife Ecology

^2^ Wildlife Conservation Society, Bronx, New York, USA

***Correspondence:**

Mag. med. vet. Nina Eva Trimmel

nina.trimmel@gmail.com

**Supplementary Table 1.** Notifiable epizootics in Austria according to the §16 Federal law on epizootics. (Tierseuchengesetz − TSG. 2019. §16 TSG) (1).

| - 1. | - Rabies^1,4^ | - 19. | - Aujeszky's disease in domestic pigs^1,4,5^; |
| --- | --- | --- | --- |
| - 2. | - Foot and Mouth disease^4^ | - 20. | - Glanders^4^ |
| - 3. | - Anthrax^6^, blackleg^3^, *Pasteurella multocida*^?^*;* | - 21. | - Covering sickness in equines and *Equid alphaherpesvirus 3*^?^; |
| - 4. | - Contagious bovine pleuropneumonia^4^ | - 22. | - Mange of horses, donkeys, mules, hinnies, sheep and goats^1,3^; |
| - 5. | - Rinderpest^4^ | - 23. | - Equine encephalitis^1,2,7^ |
| - 6. | - Bovine tuberculosis ^1,2,3,4^ (TBC-complex) | - 24. | - Equine infectious anemia^5^ |
| - 7. | - TSE in animals (including BSE^4^ in cattle and Scrapie in sheep and goat^2,3,4,*^); | - 25. | - African horse sickness^4^ |
| - 8. | - Brucellosis in sheep and goats;^4,5^ | - 26. | - Indiana vesiculovirus^4^ |
| - 9. | - Sheeppox^4^ | - 27. | - Avian influenza^1,2^ |
| - 10. | - Bluetongue disease^4,5,6^ | - 28. | - Newcastle Disease^2,4,+^ |
| - 11. | - Rift-Valley fever^4^ | - 29. | - Fowl cholera |
| - 12. | - Lumpy Skin Disease^4,5^ | - 30. | - Psittacosis^2,3^ |
| - 13. | - Ovine rinderpest (peste des petits ruminants)^4^ | - 31. | - Viral hemorrhagic septicemia (VHS)^2^ |
| - 14. | - Classical swine fever^4^ | - 32. | - *Infectious hematopoietic necrosis virus* (IHN)^5^ |
| - 15. | - African swine fever^4^ | - 33. | - *Salmon isavirus* (ISA)^4^ |
| - 16. | - Porcine teschovirus^3^ | - 34. | - Monkeypox^?^ |
| - 17. | - Brucellosis in pigs;^1,2^ | - 35. | - Ebola^?^ |
| - 18. | - Swine vesicular disease^4^ |  |  |

^1^ Disease mentioned in the investigated literature.

^2^ Disease still endemic to Austria or has been detected as of 2017. (2, 3)

^3^ Disease recently emerged, is sporadically occurring or has been detected between 2015 and 2019. (2, 3, 4)

^4^ Disease that Austria is currently free from as of 2019. (4, 5, 6, 7, 8, 9)

^5^ Disease that was not detected in 2017 according to the AGES Annual Veterinary Report. (2)

^6^ Currently not occurring in Austria according to the ADNS report of 2019, 1. Quarter. (10)

^7^ Equine encephalitis disease complex: Eastern equine encephalitis virus, Japanese encephalitis virus, Venezuelan equine encephalitis virus, Western equine encephalitis virus and West Nile virus. (11, 12)

^?^ No official information available.

*One emergeged case of Scrapie in a sheep in 2017 was atypical scrapie. (2, 3)

^+^ New Castle disease was detected in pigeons in 2017, but not in domestic birds or poultry. (2, 3)

**Supplementary Table 2.** Other veterinary legislation concerning compulsory disease notification (poultry diseases left out for a better overview) (13).

| **Disease** | **Law** |
| --- | --- |
| Bovine Brucellosis ^2,4^ | Bovine Health Surveillance Regulation, BGBl. II No. 334/2013* |
| Enzootic bovine leukosis ^5^ | Bovine Health Surveillance Regulation, BGBl. II No. 334/2013* |
| IBR/IPV (Infectious bovine rhinotracheitis and infectious pustular vulvovaginitis) ^6^ | Bovine Health Surveillance Regulation, BGBl. II No. 334/2013* |
| Bovine viral diarrhoea and mucosal disease (BVD/MD) ^1,3^ | BVD Regulation 2007, BGBl. II No. 178/2007 |
| Paratuberculosis in ruminants ^1,3,4^ | Paratuberculosis Ordinance, BGBl. II No. 48/2006* |
| Transmissible venereal diseases of cattle | Federal Act on Transmissible venereal diseases of cattle, Federal Law No 22/1949 |

^1^ Disease mentioned in the investigated literature.

^2^ Currently occurring in Austria according to the ADNS report of 2019, 1. Quarter. (10)

^3^ Disease still endemic to Austria or has been detectet as of 2017. (2, 3)

^4^ Disease recently emerged, is sporadically occurring or has been detected between 2015 and 2019. (2, 4)

^5^ Austria is officially declared free from EBL by the EU commission. (7)

^6^ Austria has additional guarantees for IBR/IPV and is currently free from this disease. (7)

**REFERENCES**

1. Tierseuchengesetz (TSG). *Bundesrecht konsolidiert: Gesamte Rechtsvorschrift für Tierseuchengesetz, Fassung vom 01.02.202,* Vienna (2019). Available

online at: https://www.ris.bka.gv.at/GeltendeFassung.wxe?Abfrage=BundesnormenandGesetzesnummer=10010172 (accessed May 6, 2019).

2. Herzog U, Damoser J, Höflechner-Pöltl A. *Annual Veterinary Report 2017.* Federal Ministry of Labour, Social Affairs, Health and Consumer Protection

and Austrian and Agency for Health and Food Safety (AGES), Vienna (2017).p. 1–84.

3. Federal Ministry of Labour, Social Affairs, Health and Consumer Protection and Austrian Agency for Health and Food Safety. *Zoonoses and Zoonotic*

*agents in Austria. Report 2017*. Vienna (2018). p. 1–84.

4. Kommunikationsplattform VerbraucherInnengesundheit (KVG). *Tiergesundheitsbericht Berichtszeitraum (TGB)* (2019). Available online at: https://www.verbrauchergesundheit.gv.at/tiere/krankheiten/tgb_adns/TGBMonatsberichtOesterreichMaerz2019.pdf?6y3wag (accessed May 07, 2019).

5. European Food Safety Authority (EFSA). *Chronic Wasting Disease:Addressing Risks for the EU (CWD).* (2017). Available online at: https://www.

efsa.europa.eu/en/press/news/170118 (accessed May 6, 2019).

6. Federal Ministry of Health (BMG). *Schreiben an die Landesregierungen, BMG - II/B/11 (Tierschutz, Tierseuchen- und Zoonosenbekämpfung)*

(2012). Available online at: https://www.verbrauchergesundheit.gv.at/tiere/krankheiten/schreiben_an_alle_landesregierungen_zur_info_tollwut_

ueberwa.pdf?63xzmf (accessed May 08, 2019).

7. Kommunikationsplattform VerbraucherInnengesundheit (KVG). *Freiheiten* (2016). Available online at: https://www.verbrauchergesundheit.

gv.at/tiere/krankheiten/freiheiten/freiheit.html (accessed May 07, 2019).

8. World Organisation for Animal Health (OIE). *African Swine Fever* (2018). Available online at: http://www.oie.int/en/animal-health-in-theworld/

animal-diseases/african-swine-fever/ (accessed May 6, 2019).

9. KommunikationsplattformVerbraucherInnengesundheit (KVG). *Afrikanische Schweinepest* (2018). Available online at: https://www.

verbrauchergesundheit.gv.at/tiere/krankheiten/asp_allg.html (accessed May 08, 2019).

10. KommunikationsplattformVerbraucherInnengesundheit (KVG). *Animal Disease Notification System (ADNS)* (2019). Available online at: https://

www.verbrauchergesundheit.gv.at/tiere/krankheiten/tgb_adns/ADNS_Quartalsbericht_2019_Q1.pdf?6xn2q6 (accessed May 07, 2019).

11. Hubálek Z, Halouzka J. West Nile fever - A reemerging mosquitoborne viral disease in Europe. *Emerg Infect Dis*. (1999) 5:643–50.

doi: 10.3201/eid0505.990505

12. Wikipedia*. Equine encephalitis* (2019). Available online at: https://en.wikipedia.org/wiki/Equine_encephalitis (accessed May 7, 2019).

13. KommunikationsplattformVerbraucherInnengesundheit (KVG). *Anzeigepflicht* (2019). Available online at: https://www.

verbrauchergesundheit.gv.at/tiere/krankheiten/anzeigepflichtig/anzeigepflichtig.html (accessed May 07, 2019).
